# miRNAs Alter T Helper 17 Cell Fate in the Pathogenesis of Autoimmune Diseases

**DOI:** 10.3389/fimmu.2021.593473

**Published:** 2021-04-21

**Authors:** Junxia Huang, Xinzhi Xu, Ji Yang

**Affiliations:** Department of Dermatology, Zhongshan Hospital, Fudan University, Shanghai, China

**Keywords:** miRNAs, T helper 17 cell, systemic lupus erythematosus, rheumatoid arthritis, multiple sclerosis, inflammatory bowel disease, autoimmune disease

## Abstract

T helper 17 (Th17) cells are characterized by the secretion of the IL-17 cytokine and are essential for the immune response against bacterial and fungal infections. Despite the beneficial roles of Th17 cells, unrestrained IL-17 production can contribute to immunopathology and inflammatory autoimmune diseases, including multiple sclerosis, rheumatoid arthritis, and inflammatory bowel disease. Although these diverse outcomes are directed by the activation of Th17 cells, the regulation of Th17 cells is incompletely understood. The discovery that microRNAs (miRNAs) are involved in the regulation of Th17 cell differentiation and function has greatly improved our understanding of Th17 cells in immune response and disease. Here, we provide an overview of the biogenesis and function of miRNA and summarize the role of miRNAs in Th17 cell differentiation and function. Finally, we focus on recent advances in miRNA-mediated dysregulation of Th17 cell fate in autoimmune diseases.

## Introduction

Activated CD4^+^ T helper (Th) cells are critical mediators of immune response, and the dysregulation of Th cell activation and differentiation is associated with inflammatory disease. Activated Th cells can be divided into functionally distinct subsets based on their cytokine production and transcription factor expression. These subsets include Th1, Th2, Th17, and Foxp3^+^ T regulatory cells as well as the recently described T follicular helper, Th9, and Th22 cells. Specific effector functions have been described for each Th cell subset but have been challenged by the identification of Th17 cells, which secrete IL-17. Under physiological conditions, Th17 cells accumulate at the mucosal surfaces of the gut, skin, and lung where they exert a protective immune function (1). Elimination of Th17 cells, but not Th1 cells, prevents the development of experimental autoimmune encephalomyelitis (EAE), an autoimmune disease of the central nervous system (2). We now know that Th17 cells are involved in nearly all major autoimmune diseases, including multiple sclerosis (MS), rheumatoid arthritis (RA), inflammatory bowel disease (IBD), and systemic lupus erythematosus (SLE) (3). Due to the strong association of Th17 cells with autoimmune diseases, there is a substantial interest in understanding the divergent functions of Th17 cells in homeostatic and disease states. Th17 cells are recruited to sites of inflammation by chemokine receptors, such as chemokine receptor 6 (CCR6). At the inflammation sites, Th17 cells recruit neutrophils and macrophages via secretion of cytokines, including interleukin-17A (IL-17A)/IL-17F and granulocyte-macrophage colony-stimulating factor (GM-CSF). Pro-inflammatory recruitment of neutrophils and macrophages ultimately results in tissue damage. Moreover, Th17 cells retain a degree of plasticity that enables them to co-express alternate effector cytokines, such as interferons γ (IFN-γ) or IL-10, expanding the pro-inflammatory effects of Th17 cells or change the “pathogenic” Th17 cells to “protective” Th17 cell (4–6). In addition to their role in inflammation, Th17 cells promote the maturation of B cells and play a critical role in the formation of ectopic lymphoid follicles in target organs (7, 8). The balance between Th17 cells and regulatory T cells (Treg) also emerged as an important factor in regulating autoimmunity (9). Given the strong association of Th17 cells with multiple inflammatory and autoimmune disorders, it is crucial to investigate the underlying mechanisms that balance homeostatic and pro-inflammatory Th17 cells in disease states. Improving our understanding of these mechanisms will contribute to clarify the immunologic mechanism of an autoimmune disease.

MicroRNAs (miRNAs) are a group of short, single-stranded, non-coding RNAs (10). The first miRNA was discovered in 1993 in the nematode *Caenorhabditis elegans*. These RNA molecules were reported to control the temporal development of *C. elegans* via an antisense RNA-RNA interaction (11). Today, extensive studies have elucidated the molecular functions of miRNAs and have demonstrated many key roles for miRNA in physiological and pathological processes, including metabolism, differentiation, inflammation, cancer, and immunity (12). Interference in miRNA biogenesis or deletion of specific miRNA results in a range of disease states. For example, T cell-specific deletion of Dicer, an enzyme required for microRNAs generation, leads to the reduction of CD4^+^ T cells and defective effector function of them (13). The absence of miRNAs in CD4^+^ T cells hinders the development of Treg cells. Also, CD4Cre *dicer*^Δ/Δ^ mouse can develop an inflammatory bowel disease (14). In addition, global miRNA-deficiency in CD4^+^ T cells results in a reduced IL-17 production by cytokine-stimulated Th17 cells (14, 15), implying the existence of miRNAs that specifically regulate IL-17 expression. Moreover, several miRNAs are dysregulated in an autoimmune disease and contribute to the pathogenesis of autoimmune inflammation by interfering with the differentiation and function of Th17 cells. Thus, understanding how miRNAs regulate the differentiation and effector function of Th17 cells is critical to the development of novel therapeutic strategies for inflammatory autoimmune diseases and for miRNA-based therapeutics.

In this review, we first outline the biogenesis and function of miRNA. Then, we discuss the mechanisms by which miRNAs participate in Th17 cell differentiation and function. Finally, we focus on how the dysregulation of miRNAs in Th17 cells contributes to the development of an inflammatory autoimmune disease.

## The Biogenesis and Function of miRNAs

**Figure 1:**
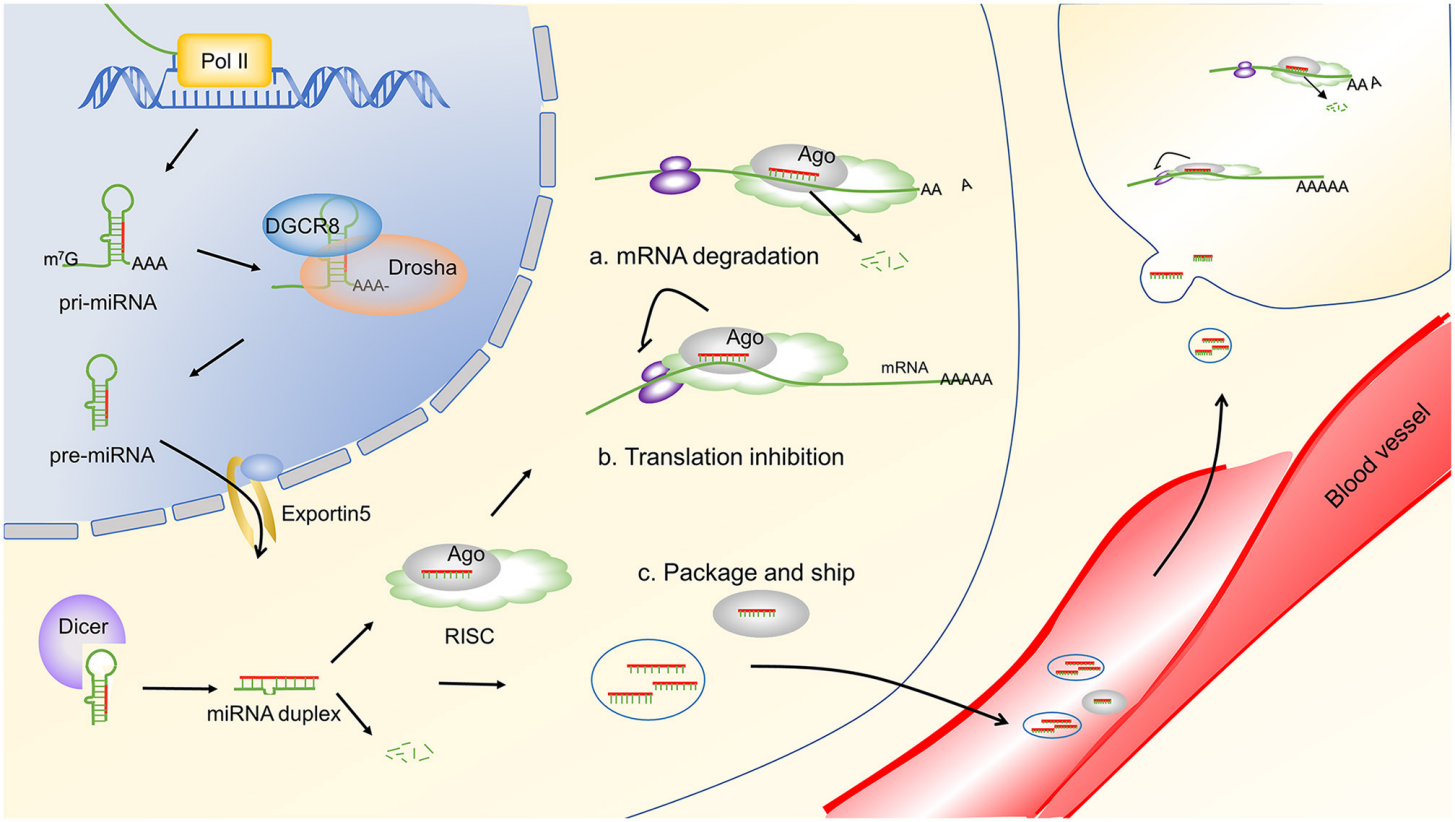
miRNA biogenesis and function. miRNA genes are transcribed by RNA polymerase II (Pol II) to generate pri-miRNAs. Then, the pri-miRNAs were cleaved to be mature miRNAs through a serious of steps. The first step is mediated by the DGCR8 complex, which generates the pre-miRNA. Then, the pre-miRNAs are exported to the cytoplasm by exportin 5. In the cytoplasm, pre-miRNAs are cleaved by Dicer to produce ~22 nucleotides of duplex miRNAs. One strand of the resulting duplex is bound by Ago proteins to form a RISC, and the other strand is commonly degraded. Finally, the mature miRNA guides the RISC to target mRNAs through complementary base pairing and represses target gene expression by either destabilizing target mRNAs **(A)** or repressing their translation **(B)**. Alternatively, miRNAs can be packaged into vesicles and transported to other cells to exert their function **(C)**. Arrow heads indicate a downstream response, and blunt lines indicate inhibition. Pol II, RNA polymerase II; m7G, 7-methylguanosine-cap; AAA, poly(A) tail; pri-miRNA, primary miRNAs; pre-miRNA, precursor miRNA; DGCR8, DiGeorge syndrome critical region gene 8; Ago, argonaute; RISC, RNA-induced silencing complex.

The biogenesis of miRNA begins with the generation of primary miRNAs (pri-miRNAs), which are typically transcribed by RNA polymerase II. The pri-miRNA transcripts are cleaved in the nucleus to form hairpin pre-miRNAs that are exported to the cytoplasm for additional processing to become ~22-nucleotide miRNA duplexes. The resulting duplexes are segregated, and individual single-strand miRNA are incorporated into the multiprotein RNA-induced silencing complex (RISC) (16–18). The functional miRNA will guide the RISC to complementary mRNA sequences, which are located primarily in the 3′ untranslated region (3′ UTR), to repress mRNA expression. In some cases, miRNAs repress target gene expression by triggering the degradation of target mRNAs and, in other cases, miRNAs repress mRNA translation at initiation or elongation stages. The biogenesis and function of miRNA are widely accepted and have been recently reviewed (19, 20). An overview of miRNA biogenesis and function is depicted in Figure 1. Importantly, individual miRNA can target multiple mRNAs, and each mRNA transcript can be recognized by multiple miRNAs. Thus, the regulation of mRNA by miRNA is extremely complex and is not fully understood. In addition to their intracellular function, miRNAs can also contribute a cell-to-cell communication via vesicle or protein carrier transportation (21–23). Secreted miRNAs can be detected in the circulation and in other bodily fluids, highlighting their potential as disease biomarkers. Moreover, miRNAs are stable in stored samples, even after periods of years, making them attractive for clinical use (24). Indeed, significant correlations have been observed between the serum or tissue miRNA profiles and disease stage or activity. For example, several miRNAs have been associated with a specific disease or treatment response after miRNA profiling in whole blood, peripheral blood mononuclear cell (PBMC), brain lesions, and synovial fluid of MS, RA, and SLE patients compared to healthy donors (25–27). Unfortunately, results have been inconsistent between studies. The source of this variation may be due to relatively small sample sizes and lack of validation in independent cohorts. Thus, large-scale studies using standardized protocols are essential to enhance the clinical development of miRNA biomarkers.

## miRNA in Th17 Cell Differentiation

miRNAs are deeply involved in both the positive and negative regulation of Th17 cell differentiation. Here, we focus on four main pathways that influence Th17 cell differentiation and are regulated by miRNA: TGF-β, janus kinase-signal transducer and activator of transcription 3 (JAK-STAT3), nuclear factor kappa-B (NF-κB), and phosphoinositide 3-kinase-protein kinase B- mammalian target of rapamycin (PI3K-AKT-mTOR).

**Figure 2:**
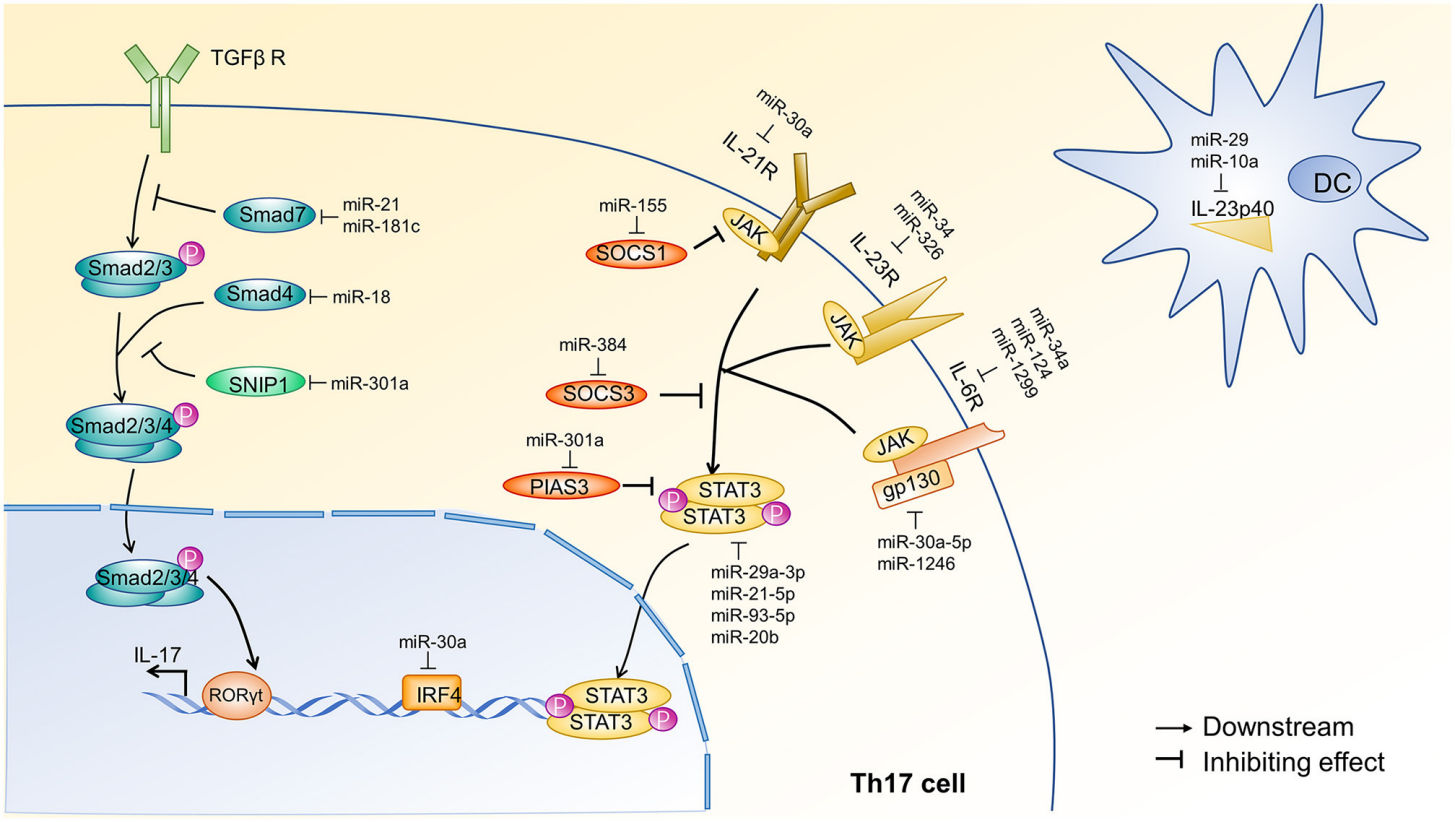
miRNA regulation of TGF-β and JAK-STAT3 signal transduction pathways during Th17 cell differentiation. The initial activation of Th17 cell differentiation is driven by the synergistic effect of JAK-STAT3 and transforming growth factor-β (TGF-β) signaling, which induce RORγt expression in naïve CD4^+^ T cells via two distinct mechanisms. JAK-STAT3 signaling is initiated by IL-6, IL-21, and IL-23. Dendritic cells (DCs) are the primary source of IL-23, and DC production of IL-23 is inhibited by miR-29 (33, 34) and miR-10 (35). Cytokine receptors on Th17 cells can also be inhibited by miRNAs. For example, miR-30a (36) inhibits IL-21R; miR-34a (37) and miR-326 (38) inhibit IL-23R; and miR-34a (37), miR-1299 (39), and miR-124 (40) inhibit IL-6R. Additionally, miR-30a-5p (39) and miR-1246 (41) target gp130, a component of the functional IL-6 family receptor complex. JAK-mediated phosphorylation of STAT3 occurs after cytokine receptor activation and is inhibited at various steps by SOCS1, SOCS3, and PIAS3. miR-155 (42), miR-384 (43), and miR-301a (44) target SOCS1, SOCS3, and PIAS3, respectively. Finally, STAT3 is directly inhibited by miR-29a-3p, miR-93-5p (45), miR-21-5p (46), and miR-20b (47). The TGF-β signal transduction pathway involves the sequential phosphorylation of Smad 2, 3, and 4, which ultimately induce IL-17 transcription. Smad7 and SNIP1 negatively regulate TGF-β signal transduction and are targeted by miRNA. Specifically, miR-21 (31) and miR-181c (32) inhibit Smad7, and miR-301a (30) inhibits SNIP1. Finally, miR-18 (29) can target Smad4. Of note, miR-30a (48) targets IRF4, another transcription factor required for the induction of ROR and IL-17A. Each of these pathways affects the expression of IL-17 in Th17 cells. Arrow heads indicate a downstream response, and blunt lines indicate inhibition. TGF-βR, transforming growth factor-β receptor; Smad, drosophila mothers against decapentaplegic protein; SNIP1, SMAD nuclear interacting protein1; ROR, Retinoid-related orphan receptor; IRF4, interferon regulatory factor4; IL-21R, Interleukin 21 receptor; IL-23R, interleukin 23 receptor; IL-6R, interleukin 6 receptor; JAK, Janus kinase; gp130, glycoprotein130; SOCS1, suppressor of cytokine signaling1; SOCS3, suppressor of cytokine signaling3; PIAS3, protein inhibitor of activated STAT3; STAT3, signal transducer and activator of transcription3; P, phosphate; DC, dendritic cell; IL-23p40, IL-12 subunit p40.

TGF-β-mediated phosphorylation of Smad2/3 is critical in the differentiation of Th17 cells. Activated Smad2/3 associate with Smad4 and translocate to the nucleus to activate the transcription (28). miR-18 inhibits Th17 cell differentiation by directly targeting Smad4 (29), whereas miR-301a enhances Th17 cell differentiation by targeting Smad nuclear interacting protein 1 (SNIP1), which competes with Smad4 to bind the coactivator CBP/P300 (30). Similarly, miR-21 (31) and miR-181c (32) induce Th17 cell differentiation via the inhibition of Smad7. Smad7 is an inhibitory SMAD protein that competitively binds to TGF-β receptor I, preventing the formation of the Smad2/3 complex and inhibiting the interaction with Smad4 (28). An overview of these mechanisms is shown in Figure 2. IL-6, IL-21, and IL-23 are cytokines that positively regulate Th17 differentiation via JAK-STAT3 signaling. Depletion of miR-29 or miR-10 in dendritic cell (DCs) promotes Th17 cell differentiation by releasing the inhibition of IL-23p40 and enhancing the production of IL-23 (33–35). Cytokine receptors on T cells activate JAK-STAT signaling to promote Th17 cell differentiation. These receptors are targeted by miRNA, ultimately influencing the differentiation of Th17 cells. Specifically, miR-34a, miR-1299, and miR-124 can target IL-6R (37, 39, 40); miR-30a targets IL-21R (36); and miR-34a and miR-326 target IL-23R (37, 38). Additionally, miR-30a-5p and miR-1246 target gp130 (39, 41), a component of the functional IL-6 family receptor complex. Cytokine receptors activate JAK, which subsequently phosphorylates STAT3. miR-29a-3p, miR-21-5p, miR-93-5p, and miR-20b impair Th17 cell differentiation by directly targeting STAT3 (45–47). In contrast, miR-155 (42), miR-384 (43), and miR-301a (44) promote the differentiation of Th17 cells through the targeted inhibition of suppressor of cytokine signaling 1 (SOCS1), suppressor of cytokine signaling 3 (SOCS3), and protein inhibitor of activated STAT3 (PIAS3), respectively. SOCS1, SOCS3, and PIAS3 are negative regulators of the JAK-STAT3 signaling pathway (49, 50). An overview of these mechanisms is shown in Figure 2.

**Figure 3:**
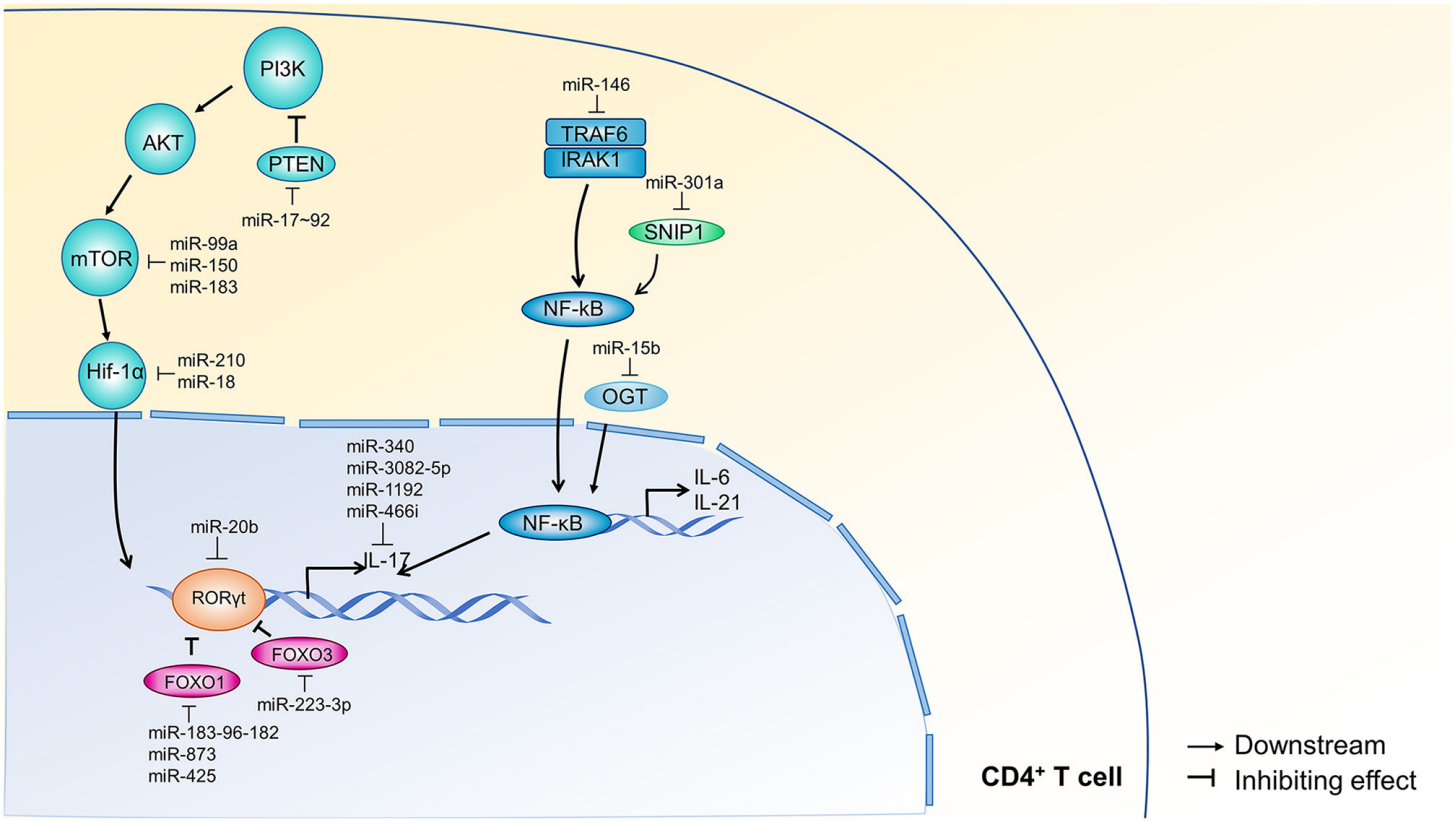
miRNA regulation of NF-κB and PI3K signal transduction pathways during Th17 cell differentiation. PI3K-AKT-mTOR-Hif-1α signaling is required for Th17 cell expansion, and NF-κB signaling contributes to the autocrine production of IL-6 and IL-21 in T cells. PI3K signals through AKT and mTOR to activate Hif-1α and downstream transcription. PTEN is a negative inhibitor of PI3K and is targeted by miR-17~92 (55). Additionally, miR-183 (56), miR-99a, and miR-150 (57) inhibit mTOR; miR-210 and miR-18 (29, 58) inhibit Hif-1α. TRAF6/IRAK1 and SNIP1 are upstream regulators of NF-κB, which contributes to the transcription of IL-6, IL-21, and IL-17. TRAF6 and IRAK1 are inhibited by miR-146 (53), and SNIP1 is inhibited by miR-301a (30). OGT also contributes to NF-κB-mediated transcription and is targeted by miR-15b (54). Within the nucleus, miR-20b (47) targets ROR, which is also inhibited by FOXO1 and FOXO3. FOXO1 is targeted by miR-183-96-182 (15), miR-873 (59), and miR-425 (60), and FOXO3 is targeted by miR-223-3p (61). IL-17 itself is inhibited by miR-340, miR-3082-5p, miR-1192, and miR466i. Arrow heads indicate a downstream response, and blunt lines indicate inhibition. PI3K, phosphoinositide 3-kinase; PTEN, phosphatase and tensin homolog deleted on chromosome ten; AKT, Ser/Thr kinase; mTOR, mammalian target of rapamycin; Hif-1α, hypoxia inducible factor 1α; ROR, retinoic acid-related orphan receptor; FOXO1, factor forkhead box protein O1; FOXO3, factor forkhead box protein O3; TRAF6, tumor necrosis factor receptor-associated factor 6; IRAK1, interleukin-1 receptor-associated kinase; SNIP1, SMAD nuclear interacting protein 1; NF-κB, nuclear factor κB; OGT, O-linked N-acetylglucosamine transferase; PCR2, polycomb repressive complex 2; JARID2, Jumonji and AT-rich interaction domain-containing 2.

NF-κB activation in response to TCR engagement is important for Th17 cell activation and differentiation by regulating the retinoic acid-related orphan receptor-γt (RORγt) (51), an important transcriptional regulator of Th17 cell differentiation (52). Tumor necrosis factor receptor-associated factor 6 (TRAF6) and interleukin 1 Receptor associated kinase 1 (IRAK1) jointly regulate NF-κB signaling in autoreactive CD4^+^ T cells. miR-146a targets TRAF6/IRAK1, blocks the NF-κB-mediated secretion of IL-6/IL-21, and inhibits the development of pathogenic Th17 cells (53). Similarly, miR-15b, an inhibitor of O-GlcNAc transferase (OGT), suppresses the differentiation of Th17 cells through an impaired O-linked N-acetylglucosamine glycosylation of NF-κB (54). Finally, miR-301a promotes Th17 cell differentiation through the downregulation of SNIP1, which suppresses the activity of NF-κB (30). An overview of these mechanisms is shown in Figure 3. Hypoxia-inducible factor 1 (Hif-1α) increases the glycolytic activity of T cells, which is crucial for rapid Th17 cell expansion (62, 63). Studies have found that miR-210 and miR-18 negatively regulate Th17 differentiation by targeting Hif-1α (29, 58). Hif-1α is activated through PI3K-AKT-mTOR signal transduction, which is targeted at several stages by miRNA. For example, miR-183, miR-99a, and miR-150 suppress Th17 cells expansion by downregulating mTOR, which is an upstream activator of Hif-1α (56, 57). In contrast, the miR-17-92 cluster represses the expression of phosphatase and tensin homology (PTEN), which antagonizes PI3K, thereby activating the PI3K-AKT-mTOR axis and facilitate Th17 cell differentiation (55, 64). An overview of these mechanisms is shown in Figure 3.

In addition to influencing these well-known Th17 cell differentiation pathways, miRNA can also directly target IL-17 and its transcriptional regulators: RORγt, Forkhead box O1 (FOXO1), and Forkhead box O3 (FOXO3). miR-1192 (65), miR-3082-5p (66), miR-466i (67), and miR-340 (68) directly bind to the 3′ UTR of IL-17A mRNA and decrease the expression of IL-17A, which impedes Th17 cell differentiation. Similarly, Th17 cell differentiation is impaired by miR-20b (47) inhibition of RORγt. FOXO1 and FOXO3 are negative regulators of the ROR transcription factors and are targeted by several miRNAs to promote Th17 differentiation (15, 59, 60). An overview of these mechanisms is shown in Figure 3.

## miRNA-Mediated Th17 Cell Abnormality in Autoimmune Diseases

As discussed above, miRNAs modulate the expression of many regulatory proteins that are required for the normal development and function of Th17 cells. Moreover, aberrant expression of miRNAs contributes to the pathology of autoimmune disease through various mechanisms (25, 69, 70). In this section, we focus on how miRNAs regulate Th17 cell differentiation to influence autoimmune diseases, including MS, RA, IBD, and SLE.

### miRNAs in MS

Multiple sclerosis (MS) is a chronic, inflammatory, demyelinating, and neurodegenerative disease of the central nervous system (CNS) in young adults. Evidence points to the IL-23-Th17 cell axis as a major inducer of CNS inflammation (71). Indeed, IL-23p19^−/−^ mice, IL-17A^−/−^ mice, and mice treated with anti-IL-17A are resistant to the induction of experimental autoimmune encephalomyelitis (EAE), a mouse model of MS (72, 73). As IL-17 and IL-22 disrupt tight junction proteins in CNS endothelial cells (74, 75), activated Th17 cells freely migrate through the blood-brain barrier into MS lesions (76). Moreover, infiltrating Th17 cells secrete GM-CSF, which recruits DCs and macrophages to promote and sustain tissue inflammation (3, 77).

The first miRNAs implicated in Th17 cell differentiation and MS development were miR-326 and miR-155. Elevated miR-326 levels are associated with IL-17A expression in CD4^+^ T cells from MS patients. Moreover, miR-326 upregulation promotes the generation of Th17 cells, but not Th1 cells, by targeting E26 avian erythroblastosis virus transcription factor-1 (Ets-1) (78). O'Connell's group reported that miR-155 is required for Th1 and Th17 differentiation during EAE development. Consistent with their findings, another study has demonstrated that miR-155 is enriched in brain-infiltrating myelin-autoreactive CD4^+^ T cells and promotes Th17 development by targeting two Hsp40 genes, DnaJ homolog subfamily member A2 (DNAJA2) and DnaJ heat shock protein family member B1 (DNAJB1) (79). Beyond its role in T cells, miR-155 is also necessary for the secretion of Th17-related cytokines in DCs (80). Other miRNAs also promote Th17 cell differentiation and exacerbate inflammatory response in EAE. For instance, miR-21 (31) and miR-181c (32) are drastically elevated in cytokine-induced-Th17 cells *in vitro*, and their upregulation promotes Th17 cell differentiation by targeting Smad7 (Figure 2). Mice deficient for miR-21 or miR-181c have a defective Th17 cell differentiation and slow EAE progression. Of note, these studies were conducted by two independent groups that used different cytokine protocols to induce Th17 cell differentiation, and the differentiation efficiency varied between the two groups. Therefore, additional studies are needed to verify that the miRNA levels are associated with Th17 cell differentiation and not with the cytokine protocol used. Moreover, whether miR-21 and miR-181c are synergistic or competitive in the promotion of Th17 cell differentiation and EAE progression remains unknown. The miR-183-96-182 (15) cluster and miR-301a are also overexpressed in T cells infiltrating the CNS in EAE. Mechanistic studies found that miR-301a activates STAT3 signaling by targeting protein inhibitors of activated STAT3 (PIAS3), an inhibitor of STAT3, ultimately promoting Th17 cell differentiation (44). Interestingly, IL-6-mediatated activation of STAT3 strongly induces miR-183-96-182 expression, which in turn inhibits FOXO1 in pathogenic Th17 cells. Similarly, STAT3 activation mediates the induction of miR-384 in CD4^+^ T cells, which reduces the expression of SOCS3, elevates the Th17/Treg ratio, and aggravates inflammation in EAE (43, 81). One final example of a positive regulation of Th17 cell differentiation by miRNA is the miR-132/212 cluster. The miR-132/212 cluster is induced by aryl hydrocarbon receptor (AHR) activation and amplifies AHR-mediated induction of Th17 cells by targeting B-cell lymphoma 6 (Bcl6) (82).

Astrocytes contribute to CNS inflammation in MS and respond to IL-17 by releasing chemokines and pro-inflammatory cytokines that recruit Th17 cells to the site of inflammation. Liu's group reported that miR-409-3p, miR-1896, and miR-873 are upregulated in IL-17 stimulated astrocytes and target SOCS3 and A20. Upregulation of these miRNAs further induces the production of inflammatory cytokines and chemokines, such as IL-1β, IL-6, IP-10, and MCP-1, to recruit pathogenic CD4^+^ T cells to the CNS, ultimately aggravating EAE development (83, 84).

miRNAs also negatively regulate Th17 cell differentiation and protect against inflammatory responses in MS. For example, miR-146a is an important negative regulator of the inflammatory response. Mice deficient for miR-146a develop a more severe EAE featuring an exaggerated Th17 cell response to autoantigens due to an enhancement of the IL-6/IL-21 pathway in CD4^+^ T cells. Although miR-146a is a negative feedback regulator of NF-κB signaling, expression of miR-146a is induced by NF-κB. In turn, miR-146a targets TRAF6 and IRAK1 to repress NF-κB signaling (53). Additionally, miR-30a and miR-20b are downregulated in differentiated Th17 cells and in CD4^+^ T cells from MS patients and EAE mice. Mice infected with lentivirus-mediated-miR-30a overexpression or lentivirus-mediated-miR-20b overexpression exhibit defective Th17 cell differentiation and slow EAE progression. Disulfiram, diphenhydramine hydrochloride, and toll like receptor 4 (TLR4) activation increase miR-30a expression, which in turn targets IRF4 and IL-21R, resulting in a suppressed Th17 cell differentiation (36, 48, 85). miR-20b also suppresses Th17 cell differentiation by directly inhibiting STAT3 and ROR (47). As miRNAs that promote or inhibit Th17 cell differentiation in MS can exist in the same cell type or even within the same cell, the coordination and regulation of miRNA signaling is a crucial question. Future research should address the network regulation of miRNAs in the same cells and how this regulation contributes to the pathogenesis of MS. A systematic analysis using genomics and deep-sequencing approaches is necessary to clarify this complex regulatory network.

### miRNAs in RA

Rheumatoid arthritis (RA) is a systemic autoimmune disease characterized by a chronic inflammation of the synovial tissues in joints associated with bone and cartilage damage. In RA patients, IL-17 levels are elevated in the synovial fluid and synovial tissue. Th17 cells predominantly express CC chemokine receptor 6 (CCR6) and migrate to joints in response to CC chemokine ligand 20 (CCL20), the ligand of CCR6, which is expressed in synoviocytes (86, 87). The aggregation of Th17 cells is an important early event in joint inflammation and is followed by a series of inflammatory amplification events, including fibroblast-like synoviocyte activation, synovial-resident innate lymphoid cell expansion, osteoclast differentiation, and neutrophil infiltration (88–92).

Similar to MS, miR-155 is upregulated in synovial fluid (SF) macrophages of RA patients. Overexpression of miR-155 increases the level of pro-inflammatory cytokines and drives Th17 cell differentiation and function by directly targeting Src homolog 2-containing inositol phosphatase-1 (SHIP-1). *In vivo*, miR-155 knockout results in the profound suppression of antigen-specific Th17 cells and relieves collagen-induced arthritis (93).

Additionally, the imbalance between Th17 and Treg cell populations is known to be involved in RA pathogenesis. RA patients have elevated levels of Th17 cells and reduced levels of Treg cells in PBMC, which results in a high Th17/Treg ratio. Although miR-21 levels are negatively correlated with the Th17/Treg ratio in PBMC from RA patients (94), the function of miR-21 in T cells has not been studied. In contrast, miR-34 elevates the Th17/Treg ratio (95, 96) and is regulated by GM-CSF and TLR ligands in blood DCs from patients with RA. Moreover, miR-34 drives DC activation and inflammatory cytokine production by targeting AXL, a critical molecular switch that inhibits TLR ligand-induced DC activation (95). In CD4^+^ T cells from RA patients, miR-34a expression is induced by IL-6 and TNF-α through the NF-κB signaling pathway. Furthermore, upregulation of miR-34a disrupts the Th17/Treg balance by targeting FOXP3 and contributes to the progress of collagen-induced arthritis in mice (96). Interestingly, IL-6 and TNF-α can also induce miR-10b-5p expression, which dampens IL-17 production by targeting MAP3K7 (97). Similar to MS, there is a complex regulatory network that connects inflammatory cytokines and miRNAs in RA.

### miRNAs in IBD

Inflammatory Bowel Disease (IBD) is described as chronic inflammatory disorders of the gastrointestinal tract, including Crohn's disease (CD) and ulcerative colitis (UC). Although Th17 cells provide a protective effect in the gut under steady states, they are also a chief pathogenic contributor in IBD (67, 98). Most Th17 cells reside in mucosal surfaces to stop the invasion of pathogens and to facilitate intestinal barrier integrity (99, 100). However, pathogenic Th17 profiles have been found in patients with IBD (98, 101, 102). Indeed, transferring the microbiota of IBD patients into germ-free mice increases intestinal Th17 cells and exacerbates colitis (103).

Individual miRNA can exert similar or opposing effects depending on the inflammatory disorder. For instance, expression of miR-301a is elevated in MS as well as in the inflamed mucosa and PBMC of CD and UC patients. In IBD, pro-inflammatory cytokines, like TNF-α, IL-23, and IL-6, enhance miR-301a expression in CD4^+^ T cells, which in turn facilitate Th17 cell differentiation and TNF-α production by inhibiting SNIP1. Downregulation of miR-301a *in vivo* alleviates 2,4,6-trinitrobenzene sulfonic acid (TNBS)-induced colitis in mice (30). In contrast, miR-34a has a potent pro-inflammatory role in RA but is a safeguard for the inflammatory stem cell niche and reparative regeneration in *Citrobacter rodentium*-induced colitis in mice. Wang's group demonstrated that miR-34a inhibits Th17 cell differentiation, expansion, and recruitment by targeting IL-6R, IL-23R, and CCL22. Moreover, they found that miR-34a restrains inflammation-induced stem cell proliferation by targeting interleukin-17 receptor D (IL-17RD) (37). It is important to note that these studies were performed in mouse and Jurkat cells, and validation of the role of miR-34a in human inflammatory mucosa is still necessary.

Data from a mouse model of IBD suggest that the development of disease requires the transition of a subset of Th17 cells to Th1-like cells that express IFN-γ (4, 104). miR-340 facilitates the pathogenic “Th1-like” differentiation of a subset of Th17 cells in human CD4^+^ T cells (105) whereas miR-210 inhibits this conversion (58). Wang et al. demonstrated that miR-210 inhibits Th17 cell differentiation during reoxygenation by targeting Hif-1α. Specifically, mice with miR210^−/−^ T cells have elevated levels of Th17 cells, enhanced conversion of Th17 cells to “Th1-like” cells, and more severe disease in an adoptive transfer colitis model (58). These studies suggest that the pathogenic transformation of Th17 cells may contribute to IBD.

Dendritic cells are potent supporters of Th17 cells differentiation because they secrete many of the cytokines required for Th17 polarization. miR-29 and miR-10a are markedly downregulated in DCs from IBD patients due to nucleotide-binding oligomerization domain 2 (NOD2) polymorphisms and elevated TNF-α, respectively. Both miR-29 and miR-10a inhibit IL-23-driven Th17 and IL-12-driven Th1 cells responses by targeting IL-12/IL-23p40, which downregulates mucosal inflammation. Moreover, miR-10a also targets NOD2 in DCs. Therefore, the inter-regulation of miR-10a and miR-29 needs to be further explored (34, 35). Finally, miR-125 and miR-219 are downregulated in CD4^+^ T cells from the peripheral blood and inflamed mucosa of IBD patients. Lentivirus-mediated upregulation of miR-125 or miR-219a-5p in CD4^+^T cells inhibits both IL-17A and IFN-γ production (106, 107). The pathogenicity of mucosal Th17 cells is not only defined by IL-17 secretion but also their plastic nature in IBD. The role of miRNAs in the conversion of Th17 to Th1-like cell needs further investigation to better understand IBD pathogenesis.

### miRNAs in SLE

Systemic lupus erythematosus (SLE) is an autoimmune disease that affects almost every organ in the body and is characterized by protean clinical manifestations. Similar to other inflammatory autoimmune diseases, pathogenic functions of Th17 cells have been reported in SLE. Th17 cells and IL-17 levels are elevated in PBMC and various tissues from patients with active SLE compared to healthy donors (108–111). Consistent with the observations in RA, the percentage of Tregs is reduced in patients with active SLE, which elevates the Th17/Treg ratio. This skewed balance between Th17 cells and Treg cells contributes to the pathogenesis of SLE (108, 112, 113). Additionally, IL-17 promotes the activation of B cells and autoantibody production in SLE (114–116). Our group has demonstrated that IL-17 increases the expression of adhesion molecules and induces the adherence of T cells to human umbilical vein endothelial cells (HUVECs) (108). Another group found that Th17 cells contribute to atherosclerosis in SLE (117, 118). Together, these studies suggest that IL-17 and Th17 cells may contribute to a vascular injury in an active SLE.

miR-155 is involved in several inflammatory autoimmune diseases and has also been explored in SLE using miR-155-deficient mice. In MRL/lpr or pristane-induced lupus mice, miR-155 deficiency reduces IL-17, autoantibody production, and renal inflammation (119, 120). These effects are similar to those observed after the modulation of miR-155 in other inflammatory autoimmune diseases. Additionally, miR-873 is elevated in Th17 cells and in PBMC from SLE patients and facilitates the differentiation of Th17 cells through FOXO1 inhibition (59). Contrary to miR-155 and miR-873, miR-101-3p and miR-183 are reduced in patients with SLE. miR-101-3p blocks Th17 cell polarization by downregulating histone deacetylase 9 (HDAC9) in SLE (121), and miR-183 reduces Th17 cell polarization and SLE progression by targeting mTOR in MRL/lpr mice (56). Overall, the miRNAs' role in regulating Th17 cells to affect SLE development is least studied. More researches are required to explore the new miRNAs and related regulation mechanisms.

### miRNAs in Other Autoimmune Diseases

Psoriasis is a chronic, immune-mediated, inflammatory cutaneous disease. Skin biopsies taken from patients with psoriasis show a high expression of IL-17 and IL-22 (122). Furthermore, the numbers of Th17 and Th1 cells in blood and skin lesions of psoriasis patients are elevated and are positively correlated with disease activity (123, 124). Wu's group reported that an enhanced expression of miR-210 induces Th17 and Th1 cell generation in peripheral blood and skin lesions of psoriasis patients or psoriasis-like mouse models. Silencing miR-210 by systemic or topical administrating of anti-miR-210 ameliorates dermatitis and decreases the percentage of Th17 and Th1 cells in splenic cells of imiquimod (IMQ)-treated mice (125, 126). Contrary to miR-210, miR-340 is reduced in psoriasis-like mouse models. Treatment with agomir-miR-340 alleviates the severity of IMQ-induced psoriasis in mice by directly targeting IL-17A (68). The combination treatment of miR-210-antigomir and miR-340-agomir in psoriasis deserves further study.

In mice with experimental autoimmune uveitis (EAU), miR-21-5p levels increase in accordance with the proportion of Th17 cells. miR-21-5p participates in the progression of EAU by affecting the Th17/Treg ratio via the regulation of IL-10. Inhibition of miR-21-5p reduces inflammatory injuries and retinal cell apoptosis (127). miR-223-3p promotes pathogenic Th17 cell response via suppression of FOXO3. MiR-233 knockdown decreases pathogenic function of Th17 cells and ameliorates the development of EAU (61). Additionally, miR-155^−/−^ mice is also resistant to EAU due to the defective induction of Th17 and Th1 cell expansion (128).

Hashimoto's thyroiditis (HT) is a common autoimmune disease characterized by lymphocyte infiltration and thyroid follicular tissue lesion. Similar to the observations in MS patients, miR-326 is elevated in the PBMC of HT patients compared to healthy donors as well as in an iodine-induced thyroiditis NOD.H-2h4 mouse model. miR-326 promotes Th17 cell differentiation by targeting A disintegrin and metalloproteinase 17 (ADAM17) and Ets-1 in PBMC from HT patients and in a thyroiditis mouse model, respectively (38, 129).

Based on the studies discussed here, several miRNAs appear to be closely related to Th17 cell differentiation in various autoimmune diseases, such as miR-155, miR-326, and miR-21. However, the mechanisms by which they regulate Th17 cells are inconsistent. These inconsistences are likely due to inherent differences in the disease models and cells. A clear picture of miRNA-target genes has yet to be resolved and is complicated by the multiplicity of miRNA targets. A single miRNA can fine-tune the expression of hundreds of mRNA transcripts, and each mRNA can be targeted by hundreds of miRNAs. Therefore, future research should focus on a systematic analysis to identify key miRNA-target genes in various Th17 cell-dependent autoimmune diseases. Functions of miRNAs and their target genes in Th17 cells and autoimmune diseases are shown in Supplementary Table 1.

## The Clinical Utilization of miRNA in Autoimmune Diseases

The application of miRNAs in disease diagnosis and treatment is under active investigation. Recent studies have shown that various autoimmune diseases have distinct miRNAs expression profiles. A microarray analysis plus real-time quantitative PCR identified that miR-126 was specifically enriched only in the blood of the SLE patients instead of healthy donors and RA patients (130). In another study, TaqMan Low Density Arrays were used to detect 365 miRNAs in PBMC of SLE patients and healthy controls. Results showed that miR-21 was upregulated and strongly correlated with SLE disease activity (131). MiR-103a-3p elevated both in whole blood and PBMC of RA patients and asymptomatic first-degree relatives (FDRs) compared with healthy controls, and the altered miR-103a-3p expression in at risk FDRs prior to RA onset (132). In patients with MS, Keren et al. observed that a decreased expression of miR-337-3p is correlated with an increased T1/T2 lesion load, which suggested that miR-337-3p is a potential biomarker for MS disease progression. In addition, miR-199a downregulation is negatively correlated with patients with disability, and the dysregulation of miR-199a can differentiate MS from RA, several types of neurologic disorders, and healthy controls (133). These miRNAs may be potential biomarkers for autoimmune disease diagnosis. However, to date, no miRNAs have met both the high sensitivity and specificity requirements for the clinical application in an autoimmune disease. A larger sample size and other disease control groups are necessary for further verification.

Several miRNA therapies have entered human clinical trials. So far, however, no miRNA drug candidates have entered the phase III of clinical trials. An immunity related example is Cobomarsen (MRG-106), a locked nucleic acid-modified oligonucleotide inhibitor of miR-155, currently being evaluated in a phase II clinical study of patients with cutaneous T cell lymphoma (clinical trial number: NCT03837457). Here, we have detailed the influence of miR-155 in Th17 cell differentiation and in the pathogenesis of various autoimmune diseases. Based on these studies, MRG-106 may also have beneficial therapeutic applications in Th17 cell-dependent autoimmune diseases. However, several challenges must be overcome before miRNA drugs can be considered for the clinical treatment of Th17-dependent autoimmune diseases. The biggest challenge is to identify the optimal miRNA candidates in each disease type. The miRNAs identified in individual studies may be influenced by heterogeneity, confounding inclusion of additional cell types in clinical samples, and the absolute quantity of Th17 cells purified from clinical samples. Although Th17 cell-specific miRNAs have been identified in cytokine-induced-Th17 cells *in vitro*, these Th17 cells are not perfect replications of the *in vivo* Th17 cells. The induced-Th17 cell differentiation and miRNA profiles are influenced by the cytokine profiles used to polarize Th17 cells. Moreover, the identification of miRNA targets in each autoimmune disease is complicated. Fortunately, technological advances, such as single cell sequencing, may help to systematically analyze miRNA-target networks and select key miRNA target candidates. Additional challenges include designing optimal miRNA delivery vehicles that enable tissue-specific or Th17 cell-specific targeting, limiting the off-target effects and immunogenicity to a safe degree.

## Conclusion

In summary, miRNAs are important regulators in Th17 cell differentiation and contribute to the etiopathology and progression of autoimmune diseases. Regulating the expression of miRNAs may reduce or inhibit pathological Th17 cells, which is a promising therapeutic direction for autoimmune diseases. Although miRNA-based therapies are still in the early stages of investigation, numerous studies have advanced our understanding of the effects of miRNA at the pathophysiological level. Further investigation of miRNAs and their molecular mechanisms in Th17 cell-dependent autoimmune diseases will help miRNA-based therapies become a long-term clinical reality.

## Author Contributions

All authors conducted the literature review for the article and substantially contributed to the discussion of content, writing of the manuscript, and editing prior to submission.

## Conflict of Interest

The authors declare that the research was conducted in the absence of any commercial or financial relationships that could be construed as a potential conflict of interest.
