# Supplementary material for: miRNAs Alter T Helper 17 Cell Fate in the Pathogenesis of Autoimmune Diseases
Source: Front Immunol. 2021 Apr 21;12:593473. doi: 10.3389/fimmu.2021.593473 (PMC8096907; doi:10.3389/fimmu.2021.593473)
Supplement: Supplementary file 1 [file Table_1.docx]

Supplementary Material

# **Supplementary table 1:** Functions of miRNAs and their target genes in Th17 cells and autoimmune diseases

| miRNA | Target(s) | Effect(s) | Experimental system(s) | reference |
| --- | --- | --- | --- | --- |
| miRNAs promote Th17 cell | | | | |
| miR-155 | Dnaja2, Dnajb1 | Th17↑ | EAE, miR-155^-/-^ mice, transfection experiments with miRNA mimics and antagomirs in vitro | (1) |
|  | SOCS1 | Th17↑ | colitis mice model, miR-155^−/−^ mice | (2) |
|  | S1PR1 | Th17 and Tfh↑, IL-21↑ | SLE patients’ PBMC, miR-155^-/-^*Fas^lpr/lpr^* mice | (3) |
|  | SHIP | Th17↑, IFN-γ, IL-6↑ | CIA, miR-155^−/−^ mice, transfection experiments with miRNA mimics and antagomirs in vitro | (4) |
| miR-301a | PIAS3 | Th17↑ | EAE, 2D2 mice, Rag-KO mice, transfection experiments with miRNA mimics and antagomirs in vitro | (5) |
|  | SNIP1 | Th17↑, TNF-α↑ | IBD patients’ PBMC, colitis mouse model, patients’ CD4^+^ T cell transduced with LV-miR-301a and LV-anti-miR-301a | (6) |
| miR-183-96-182 | Foxo1 | Th17↑ | EAE, miR-183C^−/−^ mice, Il17f^cre^Foxo1^f/f^ mice, Rag1^−/−^ mice, 2D2 T cell | (7) |
| miR-17~92 | PTEN, IKZF4 | Th17↑ | EAE, colitis mouse model, miR-17-92^f/f^, Rag2^−/−^ mice, miR-17-92^f/f^ CD4^+^ T cell | (8) |
|  | PTEN | Tfh and Th17↑, Treg↓ | miR-17∼92^fl/fl^ mouse, colitis model of Rag1^-/-^ mice | (9) |
| miR-384 | SOCS3 | Th17↑ | EAE, Rag1^−/−^ mice, transfection experiments with LV-miRNA and LV-anti-miRNA in vivo and in vitro | (10) |
| miR-409-3p, miR-1896 | SOCS3 | Th17↑, IL‐1β and IL‐6↑ | EAE, transfection experiments with LV-miRNA and LV-anti-miRNA in vitro | (11) |
| miR-181c | Smad7 | Th17↑ | EAE, transfection experiments with LV-anti-miRNA in vivo and miRNA mimics and antagomirs in vitro | (12) |
| miR-21 | Smad7 | Th17↑ | EAE, miR-21^–/–^mice, Smad7^fl/fl^ Cd4^Cre^ mice, LNA-anti-miR-21 were administered i.v. to EAE mice | (13) |
| miR-21-5p | IL-10 | Th17↑, Treg↓ | EAU, transfection experiments with LV-anti-miRNA in vivo and in vitro | (14) |
| miR-326 | Ets1 | Th17↑ | MS patients PBMC, EAE, transfection experiments with LV-miRNA and LV-anti-miRNA in vivo and in vitro | (15) |
| miR-34a | Foxp3 | Th17↑, Treg↓, | RA and SLE patients’ PBMC, CIA, miR-34a-transgenic mice, transfection experiments with agomiRNA and antagomiRNA in vitro | (16) |
|  | AXL | Th17↑ | RA patients’ DC, CIA, miR-34a^−/−^ mice | (17) |
| miR-425 | Foxo1 | Th17↑ | colitis mice model, IBD patients’ PBMC, transfection experiments with LV-miRNA in vitro and LV-anti-miRNA in vivo | (18) |
| miR-223-3p | Foxo3 | Th17↑ | EAU, transfection experiments with miRNA mimics and inhibitor in vitro | (19) |
| miR-873 | Foxo1 | Th17↑ | MRL/lpr mice, SLE patients’ PBMC, transfection experiments with LV-miRNA and LV-anti-miRNA in vitro, LV-anti-miR-873 treatment in vivo | (20) |
|  | A20 | Th17↑, IL-6, TNF-α↑ | EAE, transfection experiments with miRNA mimics and LNA-anti-miR-873 in vitro and in vivo | (21) |
| miR-326 | ADAM17 | Th17↑ | Hashimoto's thyroiditis patients’ PBMC were transfected with miRNA mimics and inhibitors | (38) |
| miR-210 | STAT3, LYN | Th17 and Th1↑, Th2↓ | psoriasis mice model, miR-210 KO mice, Rag2^–/–^ mice, psoriasis patients’ CD4^+^ T cell and skin lesion, agomiRNA and antagomiRNA treatment in vivo | (22) |
| miRNAs inhibit Th17 cell | | | | |
| miR-210 | Hif1α | Th17↓ | Colitis mice model, miR-210^–/–^CD4^Cre^ mice, Rag^2−/−^ mice, transfection experiments with miRNA mimics in vitro | (23) |
| miR-15b | OGT | Th17↓ | EAE, MS patients PBMC, transfection experiments with miRNA mimics in vitro, agomiRNA and antagomiRNA treatment in vivo | (24) |
| miR-20b | STAT3, RORγt | Th17↓ | EAE, transfection experiments with miRNA mimics in vitro and LV-miRNA treatment in vivo | (25) |
| miR-30a | IRF4 | Th17↓ | EAE, MS patients PBMC, agommiRNA and antagomiRNA were transfected to mouse CD4^+^ T cell, LV-miRNA treatment in vivo | (26) |
|  | IL-21R | Th17↓ | EAE, LV-miRNA and LV-anti-miRNA treatment in vivo, LV-miRNA and LV-anti-miRNA transfected to mouse CD4^+^ T cell | (27) |
| miR-1299, miR-30a-5p | IL-6R, gp130 | Th17↓ | CD4^+^ T cell from umbilical cord blood, transfection experiments with miRNA mimics in vitro | (28) |
| miR-146 | TRAF6, IRAK1 | Th17↓, IFN-γ, IL-6, IL-21↓ | EAE, miR-146a^–/–^ mice, 2D2/miR-146a^–/–^ mice, RAG1^–/–^ mice | (29) |
| miR-219a-5p | ETV5 | Th17 and Th1↓ | colitis mouse model, IBD patients’ inflamed mucosa, IBD patients’ CD4^+^ T cell transfected with LV-miRNA and LV-anti-miRNA | (30) |
| miR-29-3p, miR-93-5p | T-bet, STAT3 | Th1, Th17↓ | CIA, mouse CD4^+^ T cell were transfected with miRNA mimics and inhibitors | (31) |
| miR-29 | IL-12/IL-23p40 | Th17↓ | colitis mouse model, miR-29 KO mice, DCs transfected with miRNA mimic and inhibitors | (32) |
| miR-125a | Ets1 | Th17 and Th1↓ | TNBS-induced colitis mice, miR-125a^−/−^ mice, IBD patients’ PBMC, human CD4^+^ T transfected with LV-miRNA and LV-anti-miRNA | (33) |
| miR-183 | mTOR | Th17↓, Treg↑ | MRL/lpr mice mouse CD4^+^ T cell transfected with miRNA mimics | (34) |
| miR-101-3p | HDAC9 | Th17↓ | SLE patients’ PBMC were transfected with miRNA mimics and inhibitors | (35) |
| miR-340 | IL-17A | Th17↓ | Mouse naive CD4^+^ T cell were transfected with miRNA mimics, treatment of agomir-miRNA in psoriasis mice | (36) |
| miR-18a | Smad4, Hif1a, RORa | Th17↓ | T cell-specific miR-17∼92-deficient mice, miR-18^Δ/Δ^ CD4^+^ T, miR-17∼92^Δ/Δ^Smad4^Δ/+^ T cell, mouse CD4^+^ T cell were transfected with miRNA mimics and inhibitors | (37) |
| miR-1922 | IL-17 | Th17↓ | delayed-type hypersensitivity mouse model, mouse T cell transfected with miRNA mimics and inhibitors | (38) |

EAE: experimental autoimmune encephalomyelitis; SLE: Systemic lupus erythematosus; PBMC: peripheral blood mononuclear cell; EAU: Experimental Autoimmune Uveitis; CIA: collagen induced arthritis; DC: dendritic cell; IBD: inflammatory bowel disease; KO: knockout.

1. Mycko MP, Cichalewska M, Cwiklinska H, Selmaj KW. miR-155-3p Drives the Development of Autoimmune Demyelination by Regulation of Heat Shock Protein 40. J Neurosci. 2015;35(50):16504-15.

2. Li J, Zhang J, Guo H, Yang S, Fan W, Ye N, et al. Critical Role of Alternative M2 Skewing in miR-155 Deletion-Mediated Protection of Colitis. Front Immunol. 2018;9:904.

3. Xin Q, Li J, Dang J, Bian X, Shan S, Yuan J, et al. miR-155 Deficiency Ameliorates Autoimmune Inflammation of Systemic Lupus Erythematosus by Targeting S1pr1 in Faslpr/lpr Mice. J Immunol. 2015;194(11):5437-45.

4. Kurowska-Stolarska M, Alivernini S, Ballantine LE, Asquith DL, Millar NL, Gilchrist DS, et al. MicroRNA-155 as a proinflammatory regulator in clinical and experimental arthritis. Proc Natl Acad Sci U S A. 2011;108(27):11193-8.

5. Mycko MP, Cichalewska M, Machlanska A, Cwiklinska H, Mariasiewicz M, Selmaj KW. MicroRNA-301a regulation of a T-helper 17 immune response controls autoimmune demyelination. Proc Natl Acad Sci U S A. 2012;109(20):E1248-57.

6. He C, Shi Y, Wu R, Sun M, Fang L, Wu W, et al. miR-301a promotes intestinal mucosal inflammation through induction of IL-17A and TNF-alpha in IBD. Gut. 2016;65(12):1938-50.

7. Ichiyama K, Gonzalez-Martin A, Kim BS, Jin HY, Jin W, Xu W, et al. The MicroRNA-183-96-182 Cluster Promotes T Helper 17 Cell Pathogenicity by Negatively Regulating Transcription Factor Foxo1 Expression. Immunity. 2016;44(6):1284-98.

8. Liu SQ, Jiang S, Li C, Zhang B, Li QJ. miR-17-92 cluster targets phosphatase and tensin homology and Ikaros Family Zinc Finger 4 to promote TH17-mediated inflammation. J Biol Chem. 2014;289(18):12446-56.

9. Essig K, Hu D, Guimaraes JC, Alterauge D, Edelmann S, Raj T, et al. Roquin Suppresses the PI3K-mTOR Signaling Pathway to Inhibit T Helper Cell Differentiation and Conversion of Treg to Tfr Cells. Immunity. 2017;47(6):1067-82.e12.

10. Qu X, Han J, Zhang Y, Wang Y, Zhou J, Fan H, et al. MiR-384 Regulates the Th17/Treg Ratio during Experimental Autoimmune Encephalomyelitis Pathogenesis. Front Cell Neurosci. 2017;11:88.

11. Liu X, Zhou F, Yang Y, Wang W, Niu L, Zuo D, et al. MiR-409-3p and MiR-1896 co-operatively participate in IL-17-induced inflammatory cytokine production in astrocytes and pathogenesis of EAE mice via targeting SOCS3/STAT3 signaling. Glia. 2019;67(1):101-12.

12. Zhang Z, Xue Z, Liu Y, Liu H, Guo X, Li Y, et al. MicroRNA-181c promotes Th17 cell differentiation and mediates experimental autoimmune encephalomyelitis. Brain Behav Immun. 2018;70:305-14.

13. Murugaiyan G, da Cunha AP, Ajay AK, Joller N, Garo LP, Kumaradevan S, et al. MicroRNA-21 promotes Th17 differentiation and mediates experimental autoimmune encephalomyelitis. J Clin Invest. 2015;125(3):1069-80.

14. Shi L, Guo H, Li Z, Wang Y, Wang Y, Cui Y. Adenovirus-mediated down-regulation of miR-21-5p alleviates experimental autoimmune uveoretinitis in mice. Int Immunopharmacol. 2019;74:105698.

15. Du C, Liu C, Kang J, Zhao G, Ye Z, Huang S, et al. MicroRNA miR-326 regulates TH-17 differentiation and is associated with the pathogenesis of multiple sclerosis. Nat Immunol. 2009;10(12):1252-9.

16. Xie M, Wang J, Gong W, Xu H, Pan X, Chen Y, et al. NF-kappaB-driven miR-34a impairs Treg/Th17 balance via targeting Foxp3. J Autoimmun. 2019;102:96-113.

17. Kurowska-Stolarska M, Alivernini S, Melchor EG, Elmesmari A, Tolusso B, Tange C, et al. MicroRNA-34a dependent regulation of AXL controls the activation of dendritic cells in inflammatory arthritis. Nat Commun. 2017;8:15877.

18. Yang X, He Q, Guo Z, Xiong F, Li Y, Pan Y, et al. MicroRNA-425 facilitates pathogenic Th17 cell differentiation by targeting forkhead box O1 (Foxo1) and is associated with inflammatory bowel disease. Biochem Biophys Res Commun. 2018;496(2):352-8.

19. Wei Y, Chen S, Sun D, Li X, Wei R, Li X, et al. miR-223-3p promotes autoreactive Th17 cell responses in experimental autoimmune uveitis (EAU) by inhibiting transcription factor FOXO3 expression. Faseb j. 2019;33(12):13951-65.

20. Liu L, Liu Y, Yuan M, Xu L, Sun H. Elevated expression of microRNA-873 facilitates Th17 differentiation by targeting forkhead box O1 (Foxo1) in the pathogenesis of systemic lupus erythematosus. Biochem Biophys Res Commun. 2017;492(3):453-60.

21. Liu X, He F, Pang R, Zhao D, Qiu W, Shan K, et al. Interleukin-17 (IL-17)-induced microRNA 873 (miR-873) contributes to the pathogenesis of experimental autoimmune encephalomyelitis by targeting A20 ubiquitin-editing enzyme. J Biol Chem. 2014;289(42):28971-86.

22. Wu R, Zeng J, Yuan J, Deng X, Huang Y, Chen L, et al. MicroRNA-210 overexpression promotes psoriasis-like inflammation by inducing Th1 and Th17 cell differentiation. J Clin Invest. 2018;128(6):2551-68.

23. Wang H, Flach H, Onizawa M, Wei L, McManus MT, Weiss A. Negative regulation of Hif1a expression and TH17 differentiation by the hypoxia-regulated microRNA miR-210. Nat Immunol. 2014;15(4):393-401.

24. Liu R, Ma X, Chen L, Yang Y, Zeng Y, Gao J, et al. MicroRNA-15b Suppresses Th17 Differentiation and Is Associated with Pathogenesis of Multiple Sclerosis by Targeting O-GlcNAc Transferase. J Immunol. 2017;198(7):2626-39.

25. Zhu E, Wang X, Zheng B, Wang Q, Hao J, Chen S, et al. miR-20b suppresses Th17 differentiation and the pathogenesis of experimental autoimmune encephalomyelitis by targeting RORgammat and STAT3. J Immunol. 2014;192(12):5599-609.

26. Zhao M, Sun D, Guan Y, Wang Z, Sang D, Liu M, et al. Disulfiram and Diphenhydramine Hydrochloride Upregulate miR-30a to Suppress IL-17-Associated Autoimmune Inflammation. J Neurosci. 2016;36(35):9253-66.

27. Qu X, Zhou J, Wang T, Han J, Ma L, Yu H, et al. MiR-30a inhibits Th17 differentiation and demyelination of EAE mice by targeting the IL-21R. Brain Behav Immun. 2016;57:193-9.

28. Schiavinato J, Haddad R, Saldanha-Araujo F, Baiochi J, Araujo AG, Santos Scheucher P, et al. TGF-beta/atRA-induced Tregs express a selected set of microRNAs involved in the repression of transcripts related to Th17 differentiation. Sci Rep. 2017;7(1):3627.

29. Li B, Wang X, Choi IY, Wang YC, Liu S, Pham AT, et al. miR-146a modulates autoreactive Th17 cell differentiation and regulates organ-specific autoimmunity. J Clin Invest. 2017;127(10):3702-16.

30. Shi Y, Dai S, Qiu C, Wang T, Zhou Y, Xue C, et al. MicroRNA-219a-5p suppresses intestinal inflammation through inhibiting Th1/Th17-mediated immune responses in inflammatory bowel disease. Mucosal Immunol. 2020;13(2):303-12.

31. Zhu D, Tian J, Wu X, Li M, Tang X, Rui K, et al. G-MDSC-derived exosomes attenuate collagen-induced arthritis by impairing Th1 and Th17 cell responses. Biochim Biophys Acta Mol Basis Dis. 2019;1865(12):165540.

32. Brain O, Owens BM, Pichulik T, Allan P, Khatamzas E, Leslie A, et al. The intracellular sensor NOD2 induces microRNA-29 expression in human dendritic cells to limit IL-23 release. Immunity. 2013;39(3):521-36.

33. Ge Y, Sun M, Wu W, Ma C, Zhang C, He C, et al. MicroRNA-125a suppresses intestinal mucosal inflammation through targeting ETS-1 in patients with inflammatory bowel diseases. J Autoimmun. 2019;101:109-20.

34. Li X, Luo F, Li J, Luo C. MiR-183 delivery attenuates murine lupus nephritis-related injuries via targeting mTOR. Scand J Immunol. 2019;90(5):e12810.

35. Sun H, Guo F, Xu L. Downregulation of microRNA-101-3p participates in systemic lupus erythematosus progression via negatively regulating HDAC9. J Cell Biochem. 2020.

36. Bian J, Liu R, Fan T, Liao L, Wang S, Geng W, et al. miR-340 Alleviates Psoriasis in Mice through Direct Targeting of IL-17A. J Immunol. 2018;201(5):1412-20.

37. Montoya MM, Maul J, Singh PB, Pua HH, Dahlstrom F, Wu N, et al. A Distinct Inhibitory Function for miR-18a in Th17 Cell Differentiation. J Immunol. 2017;199(2):559-69.

38. Singh NP, Singh UP, Rouse M, Zhang J, Chatterjee S, Nagarkatti PS, et al. Dietary Indoles Suppress Delayed-Type Hypersensitivity by Inducing a Switch from Proinflammatory Th17 Cells to Anti-Inflammatory Regulatory T Cells through Regulation of MicroRNA. J Immunol. 2016;196(3):1108-22.
